# High CD204^+^ tumor-infiltrating macrophage density predicts a poor prognosis in patients with urothelial cell carcinoma of the bladder

**DOI:** 10.18632/oncotarget.3887

**Published:** 2015-05-07

**Authors:** Bo Wang, Hao Liu, Xiaoliang Dong, Shaoxu Wu, Hong Zeng, Zhuowei Liu, Di Wan, Wen Dong, Wang He, Xu Chen, Limin Zheng, Jian Huang, Tianxin Lin

**Affiliations:** ^1^ Guangdong Provincial Key Laboratory of Malignant Tumor Epigenetics and Gene Regulation, Sun Yat-Sen Memorial Hospital, Sun Yat-Sen University, Guangzhou, PR China; ^2^ Department of Urology, Sun Yat-Sen Memorial Hospital, Sun Yat-Sen (Zhosngshan) University, Guangzhou, PR China; ^3^ Department of Pathology, Sun Yat-Sen Memorial Hospital, Sun Yat-Sen (Zhongshan) University, Guangzhou, PR China; ^4^ Department of Urology, Cancer Center, Sun Yat-Sen (Zhongshan) University, Guangzhou, PR China; ^5^ State Key Laboratory of Oncology in South China, Cancer Center, Sun Yat-Sen (Zhongshan) University, Guangzhou, PR China

**Keywords:** tumor-infiltrating macrophages, CD204, CD169, urothelial cell carcinoma of the bladder (UCB)

## Abstract

Macrophages (Mφs) are a major cell type that can infiltrate solid tumors and exhibit distinct phenotypes in different tumor microenvironments. This study attempted to investigate the prognostic values of various tumor-infiltrating Mφ phenotypes in patients with urothelial cell carcinoma of the bladder (UCB), with a focus on Mφ tissue microlocalization. Mφs were assessed by immunohistochemistry in tissues from 302 UCB patients using CD68 as a pan-Mφ marker, and CD204 and CD169 as robust pro- and anti-tumoral Mφ phenotype markers, respectively. Our data showed that these Mφ phenotypes were predominately distributed in stromal (ST) rather than in intratumoral (INT) regions (all *P* < 0.0001). Surprisingly, CD204 and CD169 can be co-expressed by the same CD68^+^ Mφs. Kaplan-Meier analysis revealed that all INT- and ST-infiltrating CD204^+^ or CD169^+^ Mφ densities were inversely associated with overall survival (all *P* < 0.01). By multivariate analysis, ST-infiltrating CD204^+^ Mφ density emerged as an independent prognostic factor for overall survival (HR, 1.981; *P* = 0.022). Moreover, the density of ST-infiltrating CD204^+^ Mφs was positively associated with the tumor size (*P* = 0.001), tumor stage (*P* < 0.0001), nodal metastasis (*P* < 0.0001), and histological grade (*P* < 0.0001). Our findings suggest that CD204^+^ Mφs might play detrimental protumoral roles and represent the predominant Mφ phenotype in human bladder cancer.

## INTRODUCTION

Bladder cancer is the most common type of urological tumor and more than 90% of patients present as urothelial cell carcinoma of the bladder (UCB) [1–2]. The intravesical instillation of bacillus Calmette–Guerin has been used as an effective immunotheraputic strategy to prevent recurrence and progression in selected patients with non-muscle-invasive bladder cancer [2–4], suggesting that UCB is a potentially immunogenic type of tumor.

Tumor progression has been recognized to be the product of crosstalk that evolves between different cell types within tumors [5–6]. Tumor-infiltrating macrophages (Mφs) have been shown to have both pro- and anti-tumorigenic functions, which could be a consequence of the different tumor microenvironments that promote their differentiation into distinct subpopulations [7–8]. In mice, diverse Mφ subpopulations can be distinguished based on the expression of several specific markers [9]. However, many markers that have been used to identify murine Mφs cannot be translated to differentiate human Mφs [7, 9]. CD68, a pan-Mφ marker, is widely used to evaluate Mφ density in diverse types of human tumors; however, the expression of CD68 does not allow for discrimination between diverse Mφ phenotypes, which can be associated with different patient prognosis [10–13]. In breast cancer, CD68^+^ Mφs can be categorized into two subgroups based on thymidine phosphorylase (TP) that are associated with strikingly different prognosis: a TP^+^ Mφ group that shows a poor prognosis and a TP^−^ Mφ group that shows a good prognosis [12]. In renal cell carcinoma, combined analysis of CD11c^+^ Mφs and CD206^+^ Mφs can more accurately predict patient outcomes compared to analysis of CD68^+^ Mφs alone [13]. Therefore, a more detailed characterization of distinct Mφ subpopulations might provide an opportunity to eliminate protumoral Mφs or harness antitumoral Mφs in human cancers.

CD204, also known as scavenger receptor A, is a phagocytic pattern-recognition receptor that is primarily expressed on myeloid lineage cells and is involved in homeostatic functions, such as lipid metabolism and phagocytosis [14]. Emerging evidence has shown that the tumor microenvironment can cause the upregulation of CD204 expression on Mφs. Furthermore, a high density of tumor-infiltrating CD204^+^ Mφs is associated with worse patient outcomes in various types of cancer [15–17]. Additionally, CD169, a member of the sialic-acid-binding immunoglobulin-like lectin family, is expressed on myeloid lineage cells, notably on subpopulations of tissue resident Mφs and inflammatory Mφs in mice and humans [18–19]. A recent study showed that CD169^+^ Mφs dominate antitumor immunity by cross-presenting tumor antigens to CD8^+^ T cells [20]. Taken together, these observations suggest that CD204 and CD169 could serve as Mφ subpopulation markers and that they might aid the design of novel anti-tumor vaccines. To date, very little is known about the density, localization, and clinical relevance of CD204^+^ Mφs and CD169^+^ Mφs in human bladder cancer.

Herein, we investigated the distribution and prognostic significance of CD68^+^ Mφs, CD204^+^ Mφs, and CD169^+^ Mφs in 302 UCB patients. For all of these Mφ phenotypes, a trend for localization in stromal (ST) rather than in intratumoral (INT) regions of UCB tissues, as well as a higher frequency of ST-infiltrating CD204^+^ Mφs, could predict a poor prognosis, independent of other tumor-infiltrating Mφ phenotypic markers and clinical variables. Our data suggest that CD204^+^ Mφs can play protumoral roles that are detrimental and could represent the predominant Mφ phenotype in human bladder cancer.

## RESULTS

### Immunohistochemical characteristics

**Figure 1:**
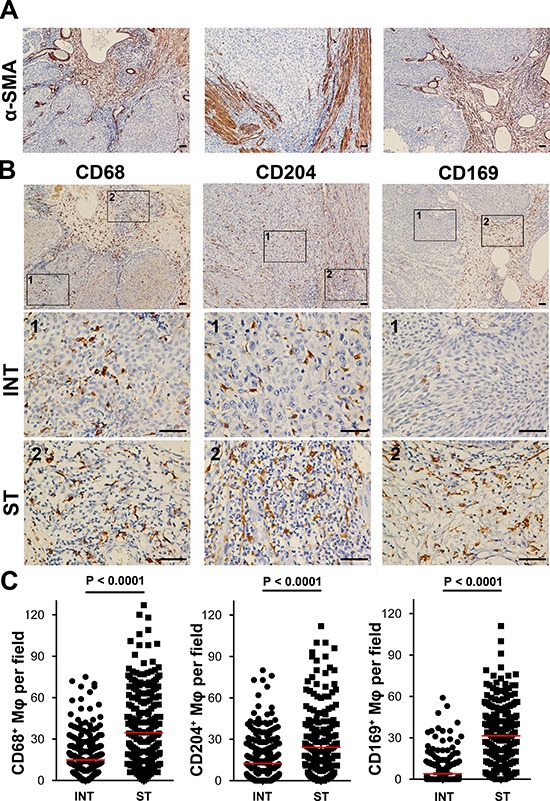
CD68+ Mφs, CD204+ Mφs, and CD169+ Mφs were enriched in the stromal regions of UCB tissues (*n* = 302) **A.** Anti-α-SMA staining was used to distinguish intratumoral (INT) from stromal (ST) regions. **B.** Representative immunohistochemistry images of CD68^+^ Mφs, CD204^+^ Mφs, and CD169^+^ Mφs in human UCB tissues. The micrographs at higher magnification show stained INT (1) and ST (2) regions. Scale bar, 100 μm. **C.** The numbers of CD68^+^ Mφs, CD204^+^ Mφs, and CD169^+^ Mφs in the INT and ST regions of human UCB tissues. Cell numbers were calculated as the cell count per × 400 field. Data are expressed as means ± SEM.

Mφs exhibit diverse phenotypes during inflammation and tumor pathogenesis [21–22]. To evaluate Mφ phenotypes and distribution patterns in human UCB tissues, we used immunostaining to study CD68^+^ Mφs, CD204^+^ Mφs, and CD169^+^ Mφs *in situ*. Clear and distinguishable staining was evident for both CD68 and each Mφ phenotype marker (Fig. 1). Using two-color immunofluorescence analyses, we also observed that CD204^+^ Mφs and CD169^+^ Mφs could be observed among the CD68^+^ Mφs (Supplementary Fig. 1).

Previous studies showed that Mφs could be detected in different locations within and around a tumor [7, 11]. We also found that CD68^+^ Mφs were distributed throughout the tissues, and were more frequent in ST than in INT regions of UCB tissues (35 ± 26 and 15 ± 14 cells/field, respectively; Fig. 1; *n* = 302). Moreover, the frequencies of both CD204^+^ Mφs and CD169^+^ Mφs were significantly higher in ST regions (24 ± 36 and 31 ± 20 cells/field, respectively) than in the corresponding INT regions of UCB tissues (13 ± 16 and 4 ± 9 cells/field, respectively). These findings indicated that CD204^+^ Mφs and CD169^+^ Mφs show the same localization bias as CD68^+^ Mφs in UCB tissues (both *P* < 0.0001; Fig. 1C).

**Figure 2:**
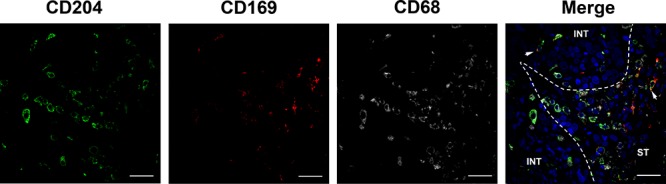
CD169 and CD204 can be co-expressed on the same Mφs in UCB tissues Paraffin-embedded UCB tissues (*n* = 5) were subjected to three-color immunofluorescence for CD204 (green), CD169 (red), and CD68 (gray), along with a DAPI counterstain (blue). While both CD204^+^ cells and CD169^+^ cells are CD68^+^ Mφs, CD204 and CD169 could colocalize on cells in both intratumoral (INT) and stromal (ST) regions (white arrows). Scale bar, 20 μm.

To analyze potential associations between cell densities for CD68^+^ Mφs, CD204^+^ Mφs, and CD169^+^ Mφs, Spearman's rank correlation coefficients were calculated (Supplementary Table 1). The densities of all three markers were positively associated with each other in INT and associated ST regions, except for the association between CD204^+^_INT_ Mφs and CD169^+^_ST_Mφs (*P* = 0.066). Using triple color immunofluorescence analyses, we also observed that CD204 and CD169 can be co-expressed on the same Mφs in both INT and ST regions of UCB tissues (Fig. 2).

### Survival analysis

**Figure 3:**
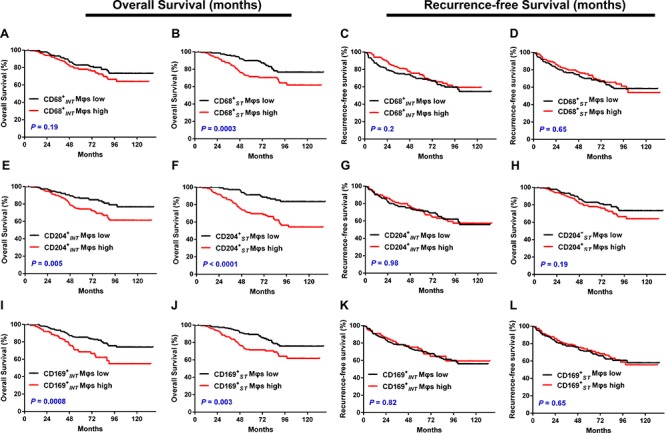
Cumulative overall survival and recurrence-free survival curves are shown for patients with UCB (*n* = 302) Kaplan–Meier survival estimates and log-rank tests were used to analyze the prognostic significance of CD68^+^ Mφs, CD204^+^ Mφs, and CD169^+^ Mφs in intratumoral (INT) and stromal (ST) regions. Patients were divided into two groups according to the median number of these Mφs per × 400 field. **A, C.** CD68^+^ Mφs in INT regions (CD68^+^_INT_ Mφs, median = 12); **B, D.** CD68^+^ Mφs in ST regions (CD68^+^_ST_ Mφs, median = 27); **E, G.** CD204^+^ Mφs in INT regions (CD204^+^_INT_ Mφs, median = 6); **F, H.** CD204^+^ Mφs in ST regions (CD204^+^_ST_ Mφs, median = 12); I, K. CD169^+^ Mφs in INT regions (CD169^+^_INT_ Mφs, median = 0); and J, L. CD169^+^ Mφs in ST regions (CD169^+^_ST_ Mφs, median = 30.5). Black lines, low group; red lines, high group.

To investigate the association of diverse Mφ phenotypes with UCB progression, we divided 302 UCB patients into two groups based on the median frequencies of CD68^+^ Mφs, CD204^+^ Mφs, and CD169^+^ Mφs, respectively. Kaplan–Meier survival curves were then plotted to further investigate associations with survival (Fig. 3). The log-rank statistic was used to compare survival rates. We did not detect any association between OS and CD68^+^_INT_ Mφ density (*P* = 0.19, Fig. 3A), but we found a marked negative association between OS and the densities of CD68^+^_ST_ Mφs (*P* = 0.0003, Fig. 3B), CD204^+^_INT_ Mφs (*P* = 0.0008, Fig. 3E), CD204^+^_ST_ Mφs (*P* = 0.003 Fig. 3F), CD169^+^_INT_ Mφs (*P* = 0.005, Fig. 3I), and CD169^+^_ST_ Mφs (*P* < 0.0001, Fig. 3J ). However, the presence of these cells did not show any prognostic significance for RFS (all *P* > 0.05, Fig. 3C–3D, 3G–3H, 3K–3L, Supplementary Table 2). After dichotomization at the median cell density for the pan-Mφ marker and Mφ markers for each activation phenotype, the 5-year OS rate was 71% above the median compared to 90% below the median for CD68^+^_ST_ Mφs, 74% vs. 87% for CD204^+^_INT_ Mφs, 70% vs. 91% for CD204^+^_ST_ Mφs, 68% vs. 85% for CD169^+^_INT_ Mφs and 71% vs. 89% for CD169^+^_ST_ Mφs. When the clinicopathological variables that were significant in the univariate analysis were adopted as covariates (Table 1), multivariate analysis revealed that the density of CD204^+^_ST_ Mφs was an independent prognostic factor for OS (HR, 1.981; *P* = 0.022).

**Table 1:** Univariate and multivariate analysis of the factors associated with overall survival for UCB

	Univariate			Multivariate		
Variable	HR	95% CI	*P*	HR	95% CI	*P*
Age, years (>60/≤60)	3.443	2.093–5.663	**1.1 × 10^−6^**	3.329	2.004–5.531	<**0.0001**
Gender (female/male)	0.672	0.309–1.46	0.317			NA
Tumor size (>3 cm/≤ 3 cm)	1.785	1.1–2.895	**0.019**	1.113	0.651–1.904	0.696
Multifocality (Multifocal/Unifocal)	0.876	0.526–1.46	0.756			NA
Tumor stage (T2–T4/Ta–T1)	3.001	1.913–4.71	**1.7 × 10**^−6^	1.915	1.071–3.422	**0.028**
Nodal metastasis (N1–N2/N0)	5.123	2.589–10.138	**2.7 × 10**^−6^	2.449	1.145–5.24	**0.021**
Histological grade (High/Low)	2.479	1.564–3.93	**1.1 × 10**^−4^	1.233	0.696–2.186	0.473
CD68^+^_INT_ Mφs (High/Low)	1.383	1.878–2.177	**0.162**			
CD68^+^_ST_ Mφs (High/Low)	2.129	1.33–3.406	**0.002**	0.91	0.527–1.572	0.735
CD204^+^_INT_ Mφs (High/Low)	1.955	1.222–3.127	**0.005**	1.042	0.59–1.84	0.887
CD204^+^_ST_ Mφs (High/Low)	3.449	2.069–5.751	**2.2 × 10**^−6^	1.981	1.101–3.564	**0.022**
CD169^+^_INT_ Mφs (High/Low)	2.145	1.358–3.389	**0.001**	1.514	0.911–2.518	0.11
CD169^+^_ST_ Mφs (High/Low)	2.009	1.26–3.202	**0.003**	1.604	0.944–2.726	0.08

**Abbreviations:** UCB, urothelial cell carcinoma of the bladder; INT, intratumoral regions; ST, stromal regions; CD68^+^_INT_ Mφs, CD68^+^ Mφs in intratumoral regions; CD68^+^_ST_ Mφs, CD68^+^ Mφs in stromal regions; HR, hazard ratio; CI, confidence interval; NA, not applicable.

**NOTE:** Univariate and multivariate analysis. Cox proportional hazards regression model. Variables associated with survival by univariate analyses were adopted as covariates in multivariate analyses. Significant *P*-values are shown in bold font.

HR > 1, risk for death increased; HR < 1, risk for death reduced.

**Figure 4:**
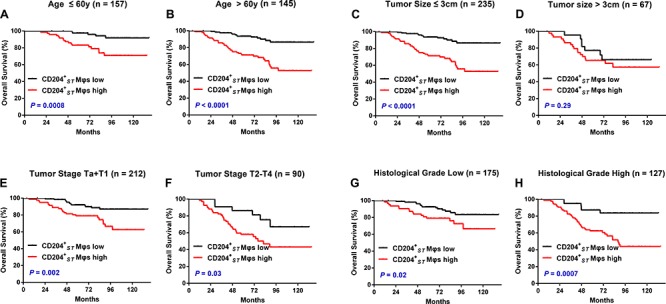
The accumulation of CD204^+^ Mφs in stromal regions predicts a poor prognosis in different subgroups of UCB patients Kaplan–Meier survival estimates and log-rank tests were used to analyze the prognostic significance of ST-infiltrating CD204^+^ Mφs (CD204^+^_ST_ Mφs) in each subgroup. All patients were stratified according to age **A–B.** tumor size **C–D.** tumor stage **E–F.** and histological grade **G–H**.

To further evaluate the prognostic value of CD204^+^_ST_ Mφs in different UCB patient subgroups, patients were stratified according to age (Fig. 4A–4B), tumor size (Fig. 4C–4D), tumor stage (Fig. 4E–4F), and histological grade (Fig. 4G–4H). The density of CD204^+^_ST_ Mφs maintained its prognostic value in predicting a shorter OS in all of these subgroups, except for OS in patients who had a tumor size greater than 3 cm (*P* = 0.29). Therefore, CD204^+^_ST_ Mφs could represent a powerful prognostic factor for patients with UCB in different risk groups.

### The association of CD204^+^_ST_ macrophage density and clinicopathological variables

The correlation between CD204^+^_ST_ Mφ density and clinicopathological variables was further evaluated by &#ξ03Χ7;&#ξ2009;^2^ analysis (Table 2). The density of CD204^+^_ST_ Mφs was positively associated with tumor size (*P* = 0.001), tumor stage (*P* < 0.0001), nodal metastasis (*P* < 0.0001), and histological grade (*P* < 0.0001). These findings indicate that large tumors, high TNM stage, and poorly differentiated UCB are risk factors that favor the infiltration of CD204^+^ Mφs in the ST of UCB tissues.

**Table 2:** Associations between CD204^+^_ST_ Mφ density and the clinicopathological characteristics of UCB

	CD204^+^_ST_ Mφ Density			
Variable	Low	High	*R*	*P*
No. of patients	151 (50%)	151 (50%)		
Age, years			0.086	0.135
≤60	85 (28%)	72 (24%)		
>60	66 (22%)	79 (26%)		
Gender			0.039	0.499
Male	133 (44%)	129 (43%)		
Female	18 (6%)	22 (7%)		
Tumor size			0.183	**0.001**
≤3 cm	129 (43%)	106 (35%)		
>3 cm	22 (7%)	45 (15%)		
Multifocality			0	1
Unifocal	107 (35%)	107 (35%)		
Multifocal	44 (15%)	44 (15%)		
Tumor stage			0.333	<**0.0001**
Ta–T1	129 (43%)	83 (27%)		
T2–T4	22 (7%)	68 (23%)		
Nodal metastasis			0.207	<**0.0001**
N0	150 (50%)	136 (45%)		
N1–N2	1 (0%)	15 (5%)		
Histological grade			0.315	<**0.0001**
Low	111 (37%)	64 (21%)		
High	40 (13%)	87 (29%)		

**Abbreviations:** UCB, urothelial cell carcinoma of the bladder; CD204^+^_ST_ Mφ, CD204^+^ Mφ in stromal regions.

Significant *P*-values are shown in bold font.

## DISCUSSION

Mφs are versatile, plastic cells that can respond to microenvironmental signals and display a broad spectrum of different phenotypes in human tumors [10, 21–22]. Therefore, we investigated the distribution of Mφ phenotypes in different microlocalizations within tumors and assessed their prognostic significance in UCB patients. We found that CD204 and CD169 are primarily expressed on CD68^+^ Mφs, and that they can be co-expressed on the same Mφs. All CD68^+^ Mφs, CD204^+^ Mφs, and CD169^+^ Mφs were found to be distributed throughout the tissue, but were often more prominent in ST than in INT regions in UCB tissues. Moreover, a high density of CD204^+^_ST_ Mφs could predict a poor prognosis for UCB patients and can be positively associated with tumor size, tumor stage, nodal metastasis, and histological grade. These data provide new insights into the significance of the location, density, and functional orientation of different immune cell populations in human tumor progression.

Previous studies have always used CD68 as a single immunohistochemical pan-Mφ marker to evaluate the location and density of Mφs and their clinical relevance in bladder cancer, but the results have been conflicting [23–24]. In a study of 63 patients with bladder cancer, Nomura *et al*. reported that patients exhibiting a high tumor-infiltrating Mφ density had worse postsurgical outcomes compared to those patients with a low tumor-infiltrating Mφ density [23]. However, in another study that used tissue microarrays to analyze 296 bladder cancer tissues, Hoglund *et al*. observed that tumor-infiltrating CD68^+^ Mφ density showed no association with patient survival [24]. These discrepancies are probably a consequence of differences in the number, stage, and size of tumors. Moreover, none of these studies evaluated the distribution of Mφ phenotypes within different microlocalizations.

Human tumor tissues can be anatomically classified into INT and ST regions, and each has distinct compositional and functional properties [25–26]. Therefore, we focused on the tissue microlocalization of CD68^+^ Mφs, CD204^+^ Mφs, and CD169^+^ Mφs in 302 patients with UCB. Our data showed that INT-infiltrating CD68^+^ Mφ density showed no association with patient survival, but ST-infiltrating CD68^+^ Mφ density was negatively associated with patient survival. In contrast to the conflicting prognistic significance of CD68^+^ Mφs (pan-Mφs), both high INT- and ST-infiltrating CD204^+^Mφ densities were found to be adverse signs in patients with UCB. Moreover, ST-infiltrating CD204^+^ Mφ density emerged as an independent predictor of prognosis and maintained its prognostic value to predict shorter survival with different risk groups in UCB patients. In accordance with our results, previous studies showed that a high density of tumor-infiltrating CD204^+^ Mφs is associated with worse prognosis in renal, lung, and pancreatic cancers [15–17]. These findings suggest that CD204^+^ Mφs could represent a stable and reliable prognostic indicator for UCB.

CD204 is cell-surface glycoprotein that belongs to the scavenger receptors with a protumoral function during tumor progression [14, 27]. Studies of mouse models showed that the expression of CD204 helped dormant tumor cells to acquire invasiveness capability, and that loss of CD204 affected tumor metastasis at the initial inoculation site [27]. Consistent with these observations, our study showed that CD204^+^ Mφs in ST regions were positively associated with tumor stage, nodal metastasis, and histological grade. Moreover, we observed that CD204 did not co-localize with immunosuppressive molecules, such as IL-10, B7-H1, or B7-H4, in UCB tissues (data not shown), which suggested that the expression of CD204 on Mφs is sufficient to promote tumor progression independently of other pathways.

CD169 is a macrophage-restricted cell surface receptor with a profound regulatory effect on T cell tumoricidal responses, which suggested that CD169^+^ Mφs are antitumorigenic [19–20]. Using a hepatocellular carcinoma model system, the antitumoral role of CD169^+^Mφs could be further supported by the finding that CD169^+^ Mφs exhibited an activated phenotype and promoted Th1/Tc1 cell responses via CD169-medicated Mφ–T cell interactions (unpublished observations). Surprisingly, we found that CD169^+^ Mφ density was associated with shorter OS in UCB, although it was not an independent predictor in multivariate analysis. As CD169 can be co-expressed with CD204 on the same Mφs, a possible explanation for the detrimental effect of Mφs on patient prognoses is that Mφ with protumoral phenotypes (e.g., CD204) might be more prevalent than those with antitumoral phenotypes (e.g. CD169) in UCB tissues. Testing this hypothesis may be the subject of further investigations.

The identification of pro- and anti-tumoral Mφ phenotypes has largely been accomplished using *in vitro* or mouse models, while less evidence has been reported for their differential expression in human tissues *in situ* [28–29]. Our findings indicated that a potent protumoral Mφ phenotype marker, CD204, and a potent antitumoral Mφ phenotype marker, CD169, can be co-expressed on the same Mφs in human UCB tissues. Moreover, the number of ST-infiltrating CD204-expressing Mφs was positively correlated with disease progression and could potentially be used as a prognostic marker for UCB patients, despite the simultaneous presence of CD169-expressing Mφs.

## MATERIALS AND METHODS

### Patients and tissue specimens

Tissue specimens were obtained from January 2003 to December 2009 from 302 patients who had pathologically confirmed UCB at the Cancer Center of Sun Yat-sen University. No patient had a distant metastasis or received anticancer therapies prior to surgery, and none of them were treated with BCG immunotherapy after surgery. All tumors were graded according to the World Health Organization 2004 classification and staged according to the TNM classification (6^th^ edition, 2002). Patient clinicopathological characteristics are summarized in Table 3. All samples were anonymously coded in accordance with the local ethical guidelines (as stipulated by the Declaration of Helsinki). Written informed consent was obtained from all patients and the protocol was approved by the Review Board of the Cancer Center.

**Table 3:** Clinicopathological characteristics of urothelial cell carcinoma of the bladder patients

Variable	No.
No. of patients	**302**
Age, years (median, range)	60 (15–90)
Gender (male/female)	262/40 (86.8%/13.2%)
Tumor size (≤3 cm/>3 cm)	235/67 (77.8%/23.2%)
Multifocality (unifocal/multifocal )	214/88 (70.9%/29.1%)
Tumor stage (Ta–T1/T2–T4)	212/90 (70.2%/29.8%)
Nodal metastasis (N0/N1–N2)	286/16 (94.7%/5.3%)
Histological grade (low/high)	175/127 (57.9%/42.1%)
Follow-up, months (median, range)	82 (4–137)

Patient follow-up data were obtained by the Cancer Center Tumor Registry, as described previously [30–32]. Briefly, patients were prospectively evaluated every 3 months during the first year, every 6 months during the second year, and annually thereafter. Follow-up visits consisted of a history, physical examination, and routine biochemical analyses. Ultrasonography of the abdomen, urography, and chest X-rays were performed at 3, 6, and 12 months postoperatively, and then annually unless otherwise clinically indicated. Abdominal/pelvic CT scans were performed 6 months postoperatively and annually thereafter. Bone scans were performed when clinically indicated. All tumor recurrences were histologically confirmed. Moreover, the survival status of all patients was updated by telephone contact in November 2014. The median follow-up for living patients was 82 months (range, 4–137 months). Overall survival (OS) was defined as the interval between surgery and death or between surgery and the last observation for surviving patients. Recurrence-free survival (RFS) was defined as the interval between surgery and recurrence or between surgery and the last observation for patients without recurrence. Among the 302 patients who were examined, 76 (25.2%) died, 100 (33.1%) had tumor recurrences, and 153 (51.7%) remained alive without recurrence during the follow-up period.

### Immunohistochemistry

Formalin-fixed and paraffin-embedded samples were cut into 5-μm sections, which then were processed for immunohistochemistry as previously described [32]. Briefly, 5-μm thick paraffin sections were first deparaffinized and hydrated, then endogenous peroxidase activity was blocked by incubating the slides in 0.3% H_2_O_2_. Antigen retrieval was performed by microwave treatment in citrate buffer (pH 6.0). Sections were blocked with normal sera from the same species from which secondary antibodies were derived. After overnight incubation at 4°C with antibodies against human α-SMA (1:500 dilution, Zhongshan Bio-Tech Co., Zhongshan, China), CD68 (1:500 dilution, Dako A/S, Glostrup, Copenhagen, Denmark), CD169 (1:200 dilution, R&D Systems, Minneapolis, MN, USA), CD204 (1:500, dilution, Transgenic, Kumamoto, Japan), or control antibodies (Santa Cruz Biotechnology, Santa Cruz, CA, USA), sections were incubated with secondary antibodies conjugated to horseradish peroxidase (Envision + Dual Link Kit, DAKO, for mouse/rabbit antibodies; or R&D Systems for donkey anti-sheep secondary antibody) for 30 min. The enzymatic reactions were developed using a peroxidase-labeled secondary antibody followed by 3,3′-diaminobenzidine tetrahydrochloride using the Envision System (Dako). Sections were counterstained with hematoxylin (Zymed Laboratories, San Francisco, CA, USA) and mounted in nonaqueous mounting medium.

### Immunofluorescence

For immunofluorescence staining, paraffin-embedded tissue sections were first incubated with rabbit anti-human CD68 and mouse anti-human CD204, or/and sheep anti-human CD169, followed by incubation with specimen-paired immunofluorescence secondary antibodies (Life Technologies). Isotype-matched primary antibodies were used as negative controls. Images were captured and analyzed on a Zeiss LSM710 system using ZEN software (Zeiss, Oberkochen, Germany).

### Evaluation of immunohistology

Tissue sections were analyzed by two independent observers who were blinded to the clinical outcome. The localization patterns of infiltrating cells in the tumors were divided into two different regions, defined as the intratumoral (INT) and stromal (ST) regions. Anti-α-SMA antibody was used to facilitate the identification of tumor ST regions, as described previously [24]. To evaluate the density of tissue-infiltrating CD68^+^ Mφs, CD169^+^ Mφs, and CD204^+^ Mφs, tissue sections were screened at a low-power field (100 ×) and the five most representative fields were selected for analysis at 400 × magnification (0.07 mm^2^ per field) using a Nikon DS-Fi2 CCD camera (Nikon, Tokyo, Japan) that was installed on a Nikon Eclipse *80i* microscope (Nikon, Tokyo, Japan). The infiltrating cells per field were enumerated manually and counts were expressed as means ± SEM. A significant linear correlation existed between the counts of two independent observers and the average count of the two investigators was used in subsequent analyses to minimize inter-observer variability.

### Statistical analyses

Statistical analyses were performed using SPSS 13.0 software (SPSS Inc., Chicago, IL, USA). The statistical significance of differences between groups was determined using the Wilcoxon signed-rank test. Cumulative survival time was calculated using the Kaplan–Meier method and was analyzed by the log-rank test. A multivariate Cox proportional hazards model was used to estimate the adjusted hazard ratios and 95% confidence intervals (CIs), and to identify independent prognostic factors. For categorical analyses, the median value was used as a cut-off to dichotomize continuous variables (for clinical applications). Associations between variables were analyzed using Spearman ρ coefficient tests and relationships between categorical variables were analyzed using *χ^2^* tests. For such comparisons, two-tailed *P*-values < 0.05 were considered to indicate statistically significant differences.

## SUPPLEMENTARY MATERIALS


