# Supplementary material for: High CD204+ tumor-infiltrating macrophage density predicts a poor prognosis in patients with urothelial cell carcinoma of the bladder
Source: Oncotarget. 2015 May 7;6(24):20204–14. doi: 10.18632/oncotarget.3887 (PMC4652998; doi:10.18632/oncotarget.3887)
Supplement: Supplementary file 1 [file oncotarget-06-20204-s001.pdf]

SUPPLEMENTARY MATERIALS

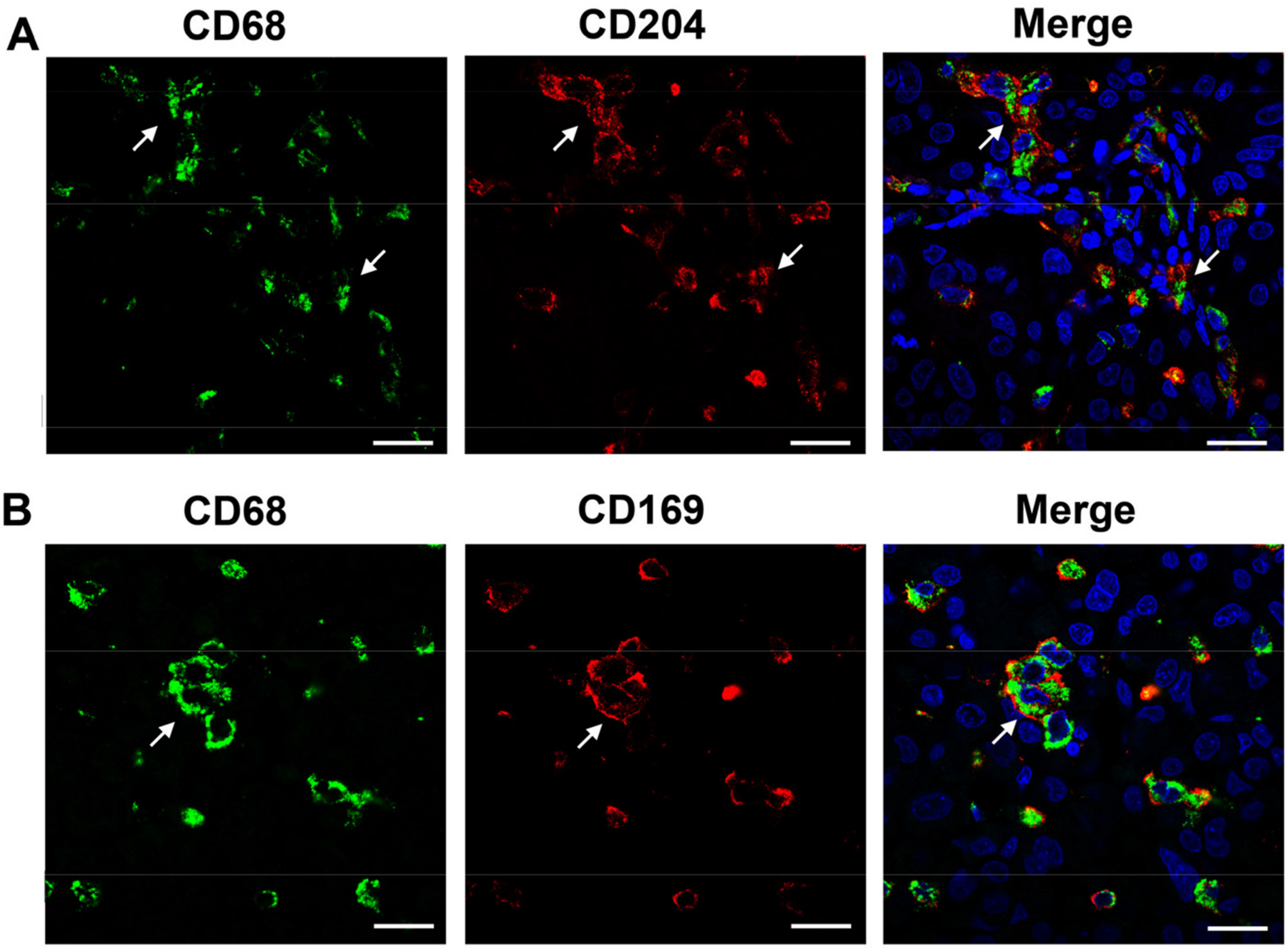

Supplementary Figure S1: CD204 and CD169 are predominately expressed by CD68+ Mφs in UCB tissues. Paraffin-embedded UCB sections (*n* = 10) were subjected to two-color immunofluorescence staining for CD68 (green) and either **A.** CD204 (red) or **B.** CD169 (red), along with a DAPI counterstain (blue). These high-power fields show the colocalization of CD68 with the indicated makers (white arrows). Scale bar, 20 µm.

Supplementary Table S1. Associations between different Mφ phenotypes in UCB tissues

	**$CD68^+_{INT}$ Mφs**	**$CD68^+_{ST}$ Mφs**	**$CD204^+_{INT}$ Mφs**	**$CD204^+_{ST}$ Mφs**	**$CD169^+_{INT}$ Mφs**	**$CD169^+_{ST}$ Mφs**
$CD68^+_{INT}$ Mφs	1	-	-	-	-	-
$CD68^+_{ST}$ Mφs	**0.001**	1	-	-	-	-
$CD204^+_{INT}$ Mφs	**5.5×10^{-7}**	**4.8×10^{-5}**	1	-	-	-
$CD204^+_{ST}$ Mφs	**0.029**	**3.8×10^{-14}**	**1.3×10^{-7}**	1	-	-
$CD169^+_{INT}$ Mφs	**3.8×10^{-6}**	**1.3×10^{-5}**	**1.4×10^{-10}**	**1.2×10^{-4}**	1	-
$CD169^+_{ST}$ Mφs	**0.021**	**4.7×10^{-12}**	0.066	**9.1×10^{-7}**	**0.019**	1

[1]χ^2 test. Significant *P*-values (< 0.05) are shown in bold font.

Abbreviations: UCB, urothelial cell carcinoma of the bladder; INT, intratumoral regions; ST, stromal regions; $CD68^+_{INT}$ Mφs, $CD68^+$ Mφs in intratumoral regions; $CD68^+_{ST}$ Mφs, $CD68^+$ Mφs in stromal regions.

Supplementary Table S2. Univariate analysis of factors associated with recurrence-free survival for UCB

	Univariate		
**Variable**	**HR**	**95% CI**	**P**
Age, years (>60/≤60)	1.354	0.912–2.011	0.132
Gender (female/male)	0.889	0.486–1.624	0.710
Tumor size (>3 cm/≤3 cm)	1.025	0.638–1.645	0.920
Multifocality (multifocal/ unifocal)	1.36	0.902–2.052	0.142
Tumor stage (T2–T4/Ta–T1)	0.929	0.597–1.445	0.745
Nodal metastasis (N1–N2/N0)	1.854	0.806–4.265	0.146
Histological grade (high/low)	1.643	1.109–2.434	**0.013**
$CD68^{+}_{INT}$ Mφs (high/low)	0.839	0.566–1.244	0.383
$CD68^{+}_{ST}$ Mφs (high/low)	0.913	0.616–1.354	0.651
$CD204^{+}_{INT}$ Mφ (high/low)	1.005	0.679–1.488	0.979
$CD204^{+}_{ST}$ Mφ (high/low)	0.977	0.658–1.449	0.906
$CD169^{+}_{INT}$ Mφs (high/low)	0.957	0.611–1.497	0.846
$CD169^{+}_{ST}$ Mφs (high/low)	0.954	0.644–1.414	0.816

Abbreviations: UCB, urothelial cell carcinoma of the bladder; INT, intratumoral regions; ST, stromal regions; $CD68^{+}_{INT}$ Mφs, $CD68^{+}$ Mφs in intratumoral regions; $CD68^{+}_{ST}$ Mφs, $CD68^{+}$ Mφs in stromal regions; HR, hazard ratio; CI, confidence interval; NA, not applicable.

NOTE: Univariate analysis. Cox proportional hazards regression model. Significant *P*-values are shown in bold. HR > 1, risk for death increased; HR < 1, risk for death reduced.
